# Genome-Wide Characterization of PIN Auxin Efflux Carrier Gene Family in *Mikania micrantha*

**DOI:** 10.3390/ijms231710183

**Published:** 2022-09-05

**Authors:** Lihua Chen, Minling Cai, Minghao Chen, Weiqian Ke, Yanru Pan, Jundong Huang, Junjie Zhang, Changlian Peng

**Affiliations:** 1 Guangzhou Key Laboratory of Subtropical Biodiversity and Biomonitoring, Guangdong Provincial Key Laboratory of Biotechnology for Plant Development, College of Life Sciences, South China Normal University, Guangzhou 510631, China; 2 Key Laboratory of Vegetation Restoration and Management of Degraded Ecosystems, South China Botanical Garden, Chinese Academy of Sciences, Guangzhou 510650, China

**Keywords:** PIN-FORMED, phylogenetic, expression profile, abiotic stress, *Mikania micrantha*

## Abstract

*Mikania micrantha*, recognized as one of the world’s top 10 pernicious weeds, is a rapidly spreading tropical vine that has invaded the coastal areas of South China, causing serious economic losses and environmental damage. Rapid stem growth is an important feature of *M. micrantha* which may be related to its greater number of genes involved in auxin signaling and transport pathways and its ability to synthesize more auxin under adverse conditions to promote or maintain stem growth. Plant growth and development is closely connected to the regulation of endogenous hormones, especially the polar transport and asymmetric distribution of auxin. The PIN-FORMED (PIN) auxin efflux carrier gene family plays a key role in the polar transport of auxin and then regulates the growth of different plant tissues, which could indicate that the rapid growth of *M. micrantha* is closely related to this PIN-dependent auxin regulation. In this study, 11 PIN genes were identified and the phylogenetic relationship and structural compositions of the gene family in *M. micrantha* were analyzed by employing multiple bioinformatic methods. The phylogenetic analysis indicated that the PIN proteins could be divided into five distinct clades. The structural analysis revealed that three putative types of PIN (canonical, noncanonical and semi-canonical) exist among the proteins according to the length and the composition of the hydrophilic domain. The majority of the PINs were involved in the process of axillary bud differentiation and stem response under abiotic stress, indicating that *M. micrantha* may regulate its growth, development and stress response by regulating PIN expression in the axillary bud and stem, which may help explain its strong growth ability and environmental adaptability. Our study emphasized the structural features and stress response patterns of the PIN gene family and provided useful insights for further study into the molecular mechanism of auxin-regulated growth and control in *M. micrantha*.

## 1. Introduction

Auxin was the earliest hormone found to promote plant growth and exists in nature mainly as indole-3-acetic acid (IAA). Auxin affects cell elongation, division and differentiation by establishing concentration gradients in different tissues [1], thereby regulating many processes of growth and development: apical dominance, root formation, inflorescence, phyllotaxy development, vascular tissue differentiation, fruit ripening and responses to light and gravity [2]. Auxin is also important for the temporal coordination of a plant’s responses to environment stresses [3,4,5]. Studies have shown that the differential distribution of the auxin concentration gradient is mainly realized by polar auxin transport (PAT), which provides a basic guarantee for plant development [6]. PAT mainly involves three kinds of auxin transport carriers, including the AUXIN RESISTENT1/LIKE AUX1 (AUX/LAX) influx carrier [7], PIN-FORMED (PIN) efflux carriers [8], and the ATP-binding cassette B/P-glycoprotein/multidrug-resistance (ABCB/MDR/PGP) efflux/condition carriers [9]. Among these auxin carriers, rearrangements of PIN locations have been observed in many critical developmental processes such as embryogenesis [10], organogenesis [11], vascular tissue development [12] and gravitropism [13], which change the direction of auxin efflux. The polar localizations of PIN proteins correspond to the direction of auxin movement, which indicates that PIN proteins are mainly responsible for the asymmetric distribution of auxin [11,14].

The PIN family, the most widely studied auxin efflux carriers, were first cloned in *Arabidopsis thaliana*, and eight AtPIN proteins were identified to form the AtPIN auxin efflux protein family [15]. According to the classification basis, *Arabidopsis* PIN proteins can be divided into different categories. Based on the subcellular localizations, one group is located in the plasma membrane (PM) and has a long central hydrophilic loop (AtPIN1-4 and AtPIN 7); another is located in the endoplasmic reticulum (ER) and the central hydrophilic loop is shorter (AtPIN5 and AtPIN8) [16,17]. In addition, the central hydrophilic loop of AtPIN6 lacks a conservative region compared to a “long” PIN; its hydrophilic loop length is between a long and short PIN; and it is found in both the PM and ER [18,19,20]. Bennett et al. (2014) proposed that PINs with a long central hydrophilic loop usually have four highly conserved regions (HC1–HC4) based on the further study of the molecular structure of PIN protein, so they are classified as “canonical” (e.g., AtPIN1-4 and AtPIN7). Short PINs lacking conserved regions are classified as “noncanonical” (e.g., AtPIN5, AtPIN8); AtPIN6 is classified as “semi- canonical” because of its special characteristics [21]. But this definition does not preclude the possibility of either a short canonical PIN protein or a long noncanonical PIN protein. A canonical PIN is mainly responsible for regulating the polar transport of auxin and joining organogenesis (AtPIN1 and 4 and 7) [11,22,23], gravitropism (AtPIN2 and 3) [24], phototropism (AtPIN1 and 3) [13,25]; the noncanonical PIN is responsible for regulating auxin homoeostasis [26] and participating in pollen development [27]; and the semi-canonical PIN has both functions because of dual localization. To sum up, the PIN gene family evolved from a common ancestor, and the difference in protein structure among its members comes mainly from the difference in the central hydrophilic loop. The functions of AtPIN protein family members in *A. thaliana* are different because of modifications to different phosphorylation sites on the central hydrophilic loop [28].

So far, PIN genes have been identified in more than 30 plant species (e.g., *A. thaliana*, *Oryza sativa*, and *Glycine max*) by genome-wide characterization [17]; however, there are few studies on the gene family members of invasive plants. Biological invasion poses a serious threat to human health, agricultural production and biodiversity. One of the world’s top 10 pernicious, invasive weeds, *M. micrantha* Kunth, is a perennial vine of the *Asteraceae* family and native to Central and South America [29]. *M. micrantha* was introduced into Hong Kong in the late 1800s and has since spread throughout southern China, resulting in significant economic losses and environmental damage [30]. *M. micrantha* grows rapidly, has a strong climbing ability [31], twines around the stem and branches of other plants, and covers the crowns of other plants, blocking sunlight [32,33], eventually leading to the loss of species diversity. Therefore, revealing the mechanism of the rapid growth of *M. micrantha* is urgently needed for research into prevention and control strategies.

One of the remarkable characteristics of the rapid growth of *M. micrantha* is the rapid elongation of its stem, which benefits from the main effect of auxin [34]. Studies have shown that compared to other Asteraceae species—sunflower (*Helianthus annuus*), lettuce (*Lactuca sativa*), artichoke thistle (*Cynara cardunculus*), chrysanthemum (*Dendranthema morifolium*) and *Artemisia annua*—*M. micrantha* has more genes involved in auxin signaling and transport pathways. Moreover, one study showed that the auxin content in the stem of *M. micrantha* after a defoliation treatment was significantly higher than that of the control group [35]. In a shading experiment, the stem of *M. micrantha* also ensured rapid growth by increasing the amount of auxin, which was beneficial for its invasion under the forest [36]. Therefore, the duplication of auxin-related genes in the *M. micrantha* genome and the response of auxin to abiotic stress indicate that the rapid growth of *M. micrantha* may be related to the regulation of auxin, which largely depends on the asymmetric distribution of the auxin polar transport system [37]. The auxin transporter PIN gene family is directly related to tissue and organ flow and the gradient distribution of auxin under normal or stress conditions [38]. Therefore, bioinformatic analysis of the *M. micrantha* PIN gene family is the primary method to further analyze the auxin regulation mechanism of *M. micrantha*. In this paper, members of the gene family were identified by bioinformatics at the whole genome level. The phylogenetic relationship, gene structure, chromosome location, cis-acting regulatory element composition, conserved motif, tissue expression profile and the abiotic stress response were analyzed to provide reference information for future studies on the biological functions of the *M. micrantha* PIN protein family. It also provided a theoretical basis to research the molecular mechanism of auxin-regulated growth and the prevention and control of *M. micrantha*.

## 2. Results

### 2.1. Identification of the PIN Gene Family of M. micrantha

After a comprehensive search, a total of 11 PIN genes were identified in the *M. micrantha* genome, which were similar to those of *A. thaliana* (8), *O. sativa* (12), and *Vitis vinifera* (8). Details about the identified genes are shown in Table 1. The sizes of the PIN protein varied from 352 to 715 amino acids; the largest PIN protein molecular weight was 77,912.95 Da (E3N88_03888), and the smallest was 38,726.75 Da (E3N88_01311). The protein isoelectric points (pIs) varied from 6.07 to 9.34. The GRAVY value was between 0.725 and −0.093: a positive value indicated that the protein was hydrophobic, a negative value indicated it was hydrophilic, and between −0.5 and 0.5 it was amphiphilic. As shown in Table 1, one PIN protein was hydrophilic (E3N88_01311); the rest were amphiphilic. The number of transmembrane regions of *M. micrantha* PIN proteins varied from 5 to 9, and the prediction of subcellular localization indicated that, except for E3N88_01311, which was located in the vascular membrane, the rest were in the plasma membrane (Table 1).

### 2.2. Phylogenetic Analysis of the PIN Gene Family of M. micrantha

**Figure 1:**
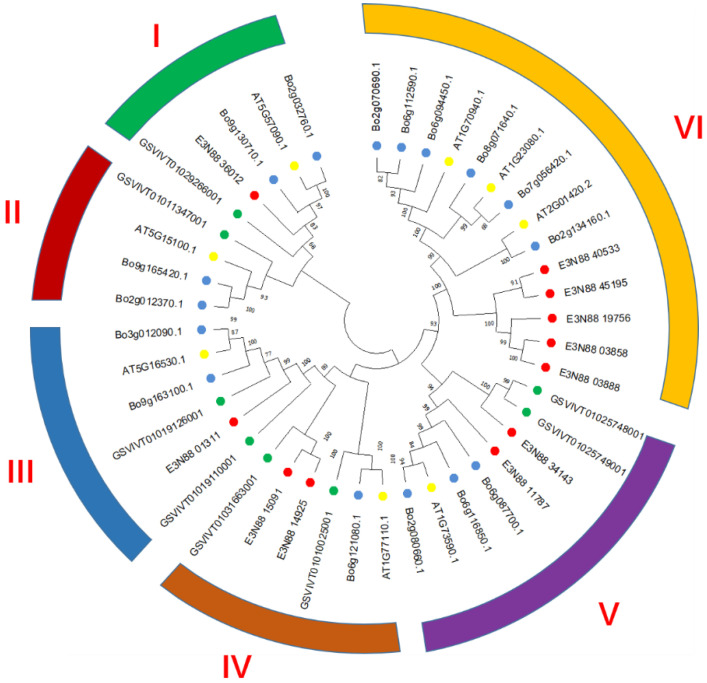
Phylogenetic analysis of PIN auxin transporter in *M. micrantha*, *A. thaliana*, *B. oleracea* and *V. vinifera*. The 43 PIN proteins from four plant species could be classified into six distinct clades (group I–VI) and differentiated in color. *M. micrantha*, *A. thaliana*, *B. oleracea* and *V. vinifera* are distinguished by solid circle shapes in red, yellow, blue and green, respectively.

Investigation of the evolutionary relationship of PIN family among *M. micrantha*, *A. thaliana*, *Brassica oleracea* and *V. vinifera* helped us to understand the possible biological functions of PINs in *M. micrantha*. From the constructed phylogenetic tree, they could be divided into six groups represented by AtPIN1, AtPIN2, AtPIN3/4/7, AtPIN5, AtPIN6, AtPIN8. They were labelled group I (PIN2), group II (PIN8), group III (PIN5), group IV (PIN6), group V (PIN1), and group VI (PIN3/4/7). Among these, one *M. micrantha* PIN belonged to group I (E3N88_36012) and group III (E3N88_01311); two belonged to group IV (E3N88_14925 and E3N88_15091) and group V (E3N88_34143 and E3N88_11787); and five belonged to group VI (E3N88_45195, E3N88_40533, E3N88_19756, E3N88_03858 and E3N88_03888). No *M. micrantha* PIN was found in group II. Protein clustering based on sequence similarity among the PINs of the four species meant that they had similar functional or subfunctional roles during species-dependent development [16]; that is, *M. micrantha* PINs and their respective groups may have had similar functions. Meanwhile, there was one paralogue gene pair (E3N88_14925, E3N88_15091) of the *M. micrantha* PIN family in group IV and two pairs (E3N88_45195, E3N88_40533 and E3N88_03858, E3N88_03888) in group VI. The five expanded *M. micrantha* PINs in group VI (E3N88_45195, E3N88_40533, E3N88_19756, E3N88_03858 and E3N88_03888) clustered with other species into two clades, and it was speculated that the possible reason was the difference among species (Figure 1).

### 2.3. Chromosome Localization of the PIN Gene Family of M. micrantha

**Figure 2:**
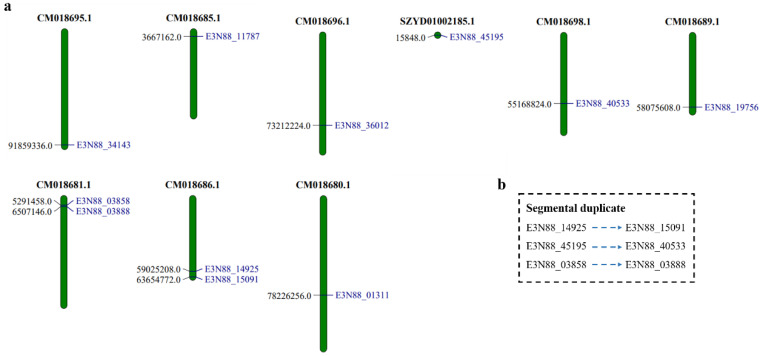
Chromosomal localization of PIN genes in *M. micrantha*. (**a**) The genome visualization tool MapChart was employed to analyze the *M. micrantha* genome. *M. micrantha* chromosomes were arranged in blocks. The 11 PIN genes were mapped by locus. (**b**) Three pairs of segmental duplicated genes E3N88_14925/E3N88_15091, E3N88_45195/E3N88_40533, E3N88_03858/E3N88_03888 are shown with dotted arrows.

Chromosome localization showed that 9 of the 11 PIN genes of *M. micrantha* were distributed on different chromosomes, whereas (E3N88_14925, E3N88_15091) and (E3N88_03858, E3N88_03888) were distributed on the same chromosome: (CM018686.1) and (CM018681.1), respectively. Gene duplication is a major contributor to evolutionary momentum [39], and plays a key role in the expansion of gene families. According to phylogenetic analysis, the *M. micrantha* PIN family shared three pairs of paralogue genes with high sequence similarity (>85%), two of which were on the same chromosome, indicating that a duplication had occurred. The three gene pairs (E3N88_14925, E3N88_15091), (E3N88_45195, E3N88_40533) and (E3N88_03858, E3N88_03888) belonged to segmental duplication according to amino acid sequence identity (Table S2) and distribution distance on chromosomes (Table 1, Figure 2).

### 2.4. Analysis of PIN Gene Structure of M. micrantha

**Figure 3:**
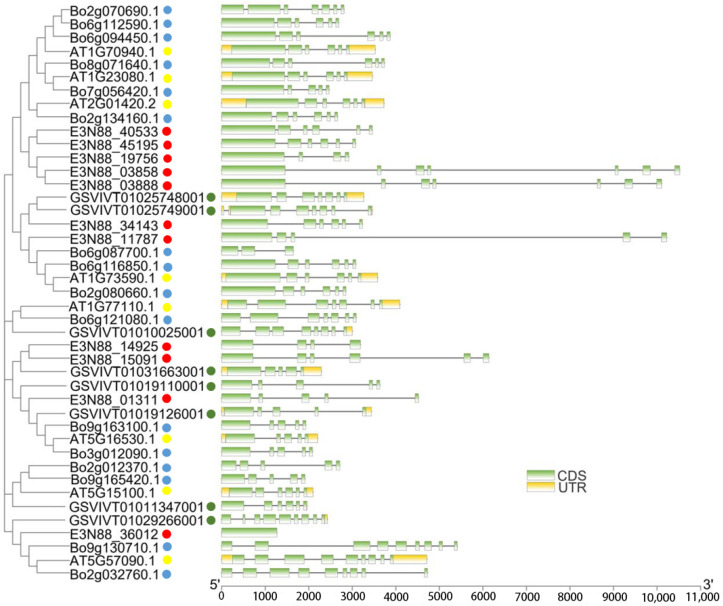
Exon–intron organization of PIN genes in *M. micrantha*, *A. thaliana*, *B. oleracea* and *V. vinifera*. Green boxes represent exons, untranslated regions (UTRs) are indicated by yellow boxes, and black lines represent introns. The lengths of the boxes and lines are scaled based on gene length. The exon and intron sizes can be estimated using the scale at the bottom. *M. micrantha*, *A. thaliana*, *B. oleracea* and *V. vinifera* are distinguished by solid circle shapes in red, yellow, blue and green, respectively.

To further understand the similarity and difference of the PIN gene structure of *M. micrantha*, the exon–intron structure of the PIN genes was analyzed by comparing the coding and genomic sequences. The results showed that the number of introns varied from three to six, except for E3N88_36012, which had no intron. The lengths of the PIN genes were quite different due to differences in intron length. In each group, except for PIN (E3N88_36012) in group I which had a gene structure quite different from that of the other three species (*A. thaliana*, *B. oleracea* and *V. vinifera*), the other PINs in the same group tended to have similar exon structure. For example, in group VI, the number of PIN exons in *M. micrantha*, *A. thaliana*, *B. oleracea* and *V. vinifera* ranged from four to seven, indicating that the gene structure of PIN members in the same group may be conserved (Figure 3).

### 2.5. Transmembrane Region Prediction, Conserved Motifs and Multiple Sequence Alignment Analysis

**Figure 4:**
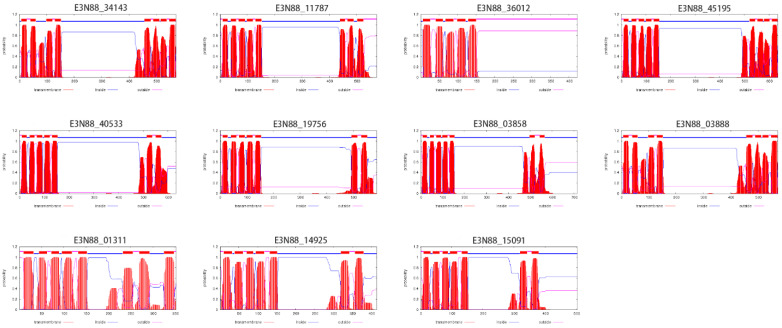
Transmembrane analysis of PIN proteins in *M. micrantha*. The red peaks represent the predicted transmembrane domain and blue color line indicate the central hydrophobic loop of proteins.

**Figure 5:**
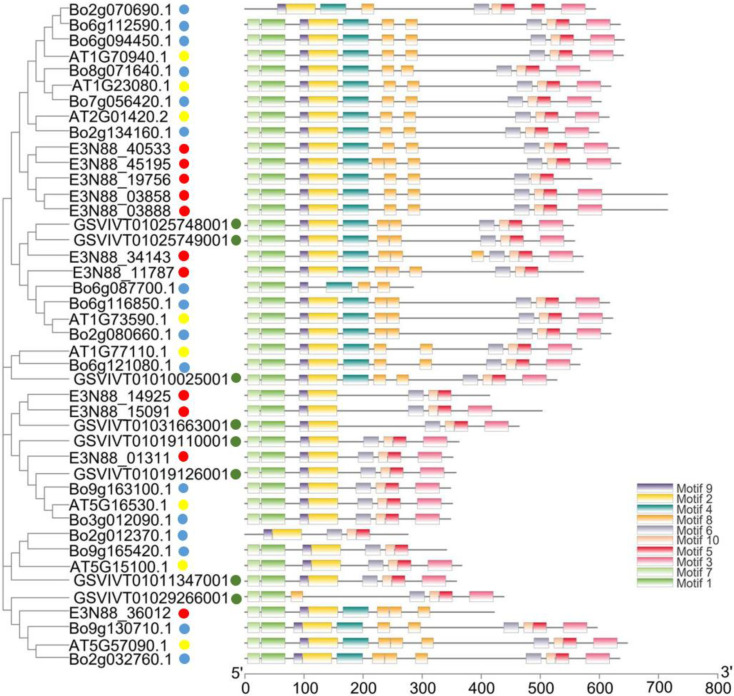
Conserved motifs analysis of PINs in *M. micrantha*, *A. thaliana*, *B. oleracea* and *V. vinifera*. Ten predicted motifs are shown by different colored boxes, and motif sizes are demonstrated by the scale on the right. *M. micrantha*, *A. thaliana*, *B. oleracea* and *V. vinifera* are distinguished by solid circle shapes in red, yellow, blue and green, respectively.

**Figure 6:**
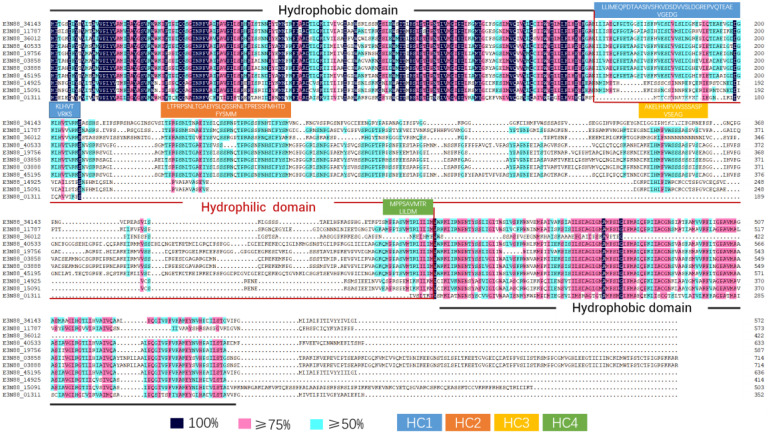
Multiple sequence alignment of PIN proteins in *M. micrantha*. Identical (100%), conservative (≥75%) and block (≥50%) of similar amino acid residues are shaded in deep blue, pink and light green, respectively. The black line indicates hydrophobic domain, the red line indicates hydrophilic domain. The motifs that constitute the highly conserved canonical regions HC1-HC4 within the common central hydrophilic region are shown as blue, orange, yellow and green rectangles, respectively.

The number of predicted transmembrane domains in *M. micrantha* PIN proteins varied from five to nine. Except for E3N88_36012, the other proteins had a typical transmembrane structure (Figure 4): two highly conserved hydrophobic transmembrane regions at the N- and C-termini and a central hydrophilic loop. The great difference in sequence length of *M. micrantha* PIN proteins (352aa–715aa) was mainly due to differences in the length of the central hydrophilic loop, the structure of which has been identified as the main functional domain of the PIN protein. According to the classification of PINs by Bennett et al. (2014) [21], a comparative analysis of the conserved motifs in the central hydrophilic loop structure of all PINs of the four species (Figure 5) and the alignment of the amino acid sequences of *M. micrantha* PINs (Figure 6) showed that most *M. micrantha* PINs have two groups of conserved motifs (1, 2, 7, 9 and 3, 5, 6, 10) at the N-terminus and C-terminus regions, respectively, and two conserved motifs (4 and 8) in the central hydrophilic loop region. At the same time, there were four highly conserved regions (HC1–HC4) in the sequence alignment results, so this type was classified as canonical PINs: (E3N88_34143, E3N88_11787, E3N88_45195, E3N88_40533, E3N88_19756, E3N88_03858 and E3N88_03888). *M. micrantha* E3N88_01311 lacked conserved motifs 4 and 8, and the conserved region of HC1–HC4 in the central hydrophilic loop, so it was classified as a noncanonical PIN. E3N88_14925 and E3N88_15091 were classified into group IV (PIN6) with *A. thaliana* AtPIN6 on the phylogenetic tree; thus, they were classified as semi-canonical PINs. The above classification was consistent with the classification results of the phylogenetic tree according to *A. thaliana* PIN characteristics. However, E3N88_36012 lacked the C-terminal transmembrane region sequence, so it cannot be classified according to the definition of a canonical PIN, but its hydrophilic region still had conserved motifs 4 and 8 and three conserved regions (HC1-HC3), which were highly similar to the *A. thaliana* AtPIN2 sequence. It was temporarily classified in the PIN2 subgroup, but its structure needs to be further identified.

### 2.6. Analysis of Cis-Acting Regulatory Elements (CAREs) of the PIN Gene Family of M. micrantha

**Figure 7:**
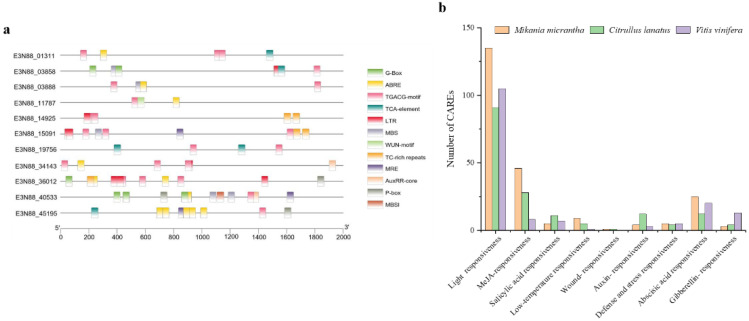
Analysis of cis-acting regulatory elements (CAREs) of the PIN gene family. (**a**) Putative CAREs of the PIN gene family in *M. micrantha*. Twelve cis-acting regulatory elements commonly found in PIN genes are shown with different colored squares, respectively. (**b**) Number of response-related CAREs of the PIN gene family in three vines.

To further understand the potential regulatory mechanism of *M. micrantha* PIN genes and how they are regulated by phytohormones and defense and stress response elements, the PlantCare web server was used to search for possible cis-elements in the 2000 bp promoter region. A total of 12 common response-related CAREs were selected in the *M. micrantha* PIN gene family, including light, methyl jasmonate (MeJA), abscisic acid, auxin, salicylic acid, defense and stress response components (Figure 7a). Among the PIN gene family of *M. micrantha*, CAREs involving light, MeJA, abscisic acid, and low temperature responses were the most prevalent, and the number of these elements was significantly higher than for the other two vines (*Citrullus lanatus* and *V. vinifera*) (Figure 7b), indicating that for *M. micrantha*, as an invasive plant, its PIN response regulation to light, low temperature, MeJA, and abscisic acid might match its strong growth and environmental adaptability.

### 2.7. Tissue Expression Profile of the PIN Gene Family of M. micrantha

**Figure 8:**
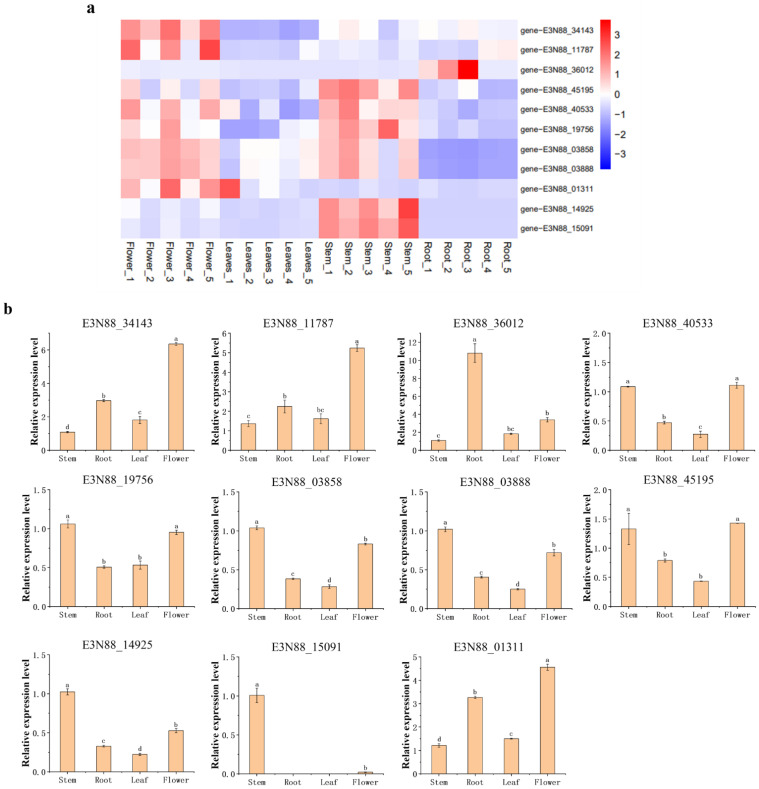
The PIN genes expression pattern in different *M. micrantha* tissues (root, stem, leaf and flower). (**a**) Expression heatmaps of PIN genes in different tissues. The FPKM values were transformed to log2 (value + 1). The color scale is shown at the right, and higher expression levels are shown in red. (**b**) Relative expressions of PIN genes in different tissues. The histogram represents the relative expression level of PIN genes in different tissues compared with the expression level in stem, which was normalized to a value of 1. The error bars represent the standard errors (SEs) of three to seven biological replicates.

The expression pattern of PIN genes in different tissues (root, stem, leaf, flower) was detected via transcriptome data and a qRT-PCR experiment (Figure 8a,b). The RNA-seq results showed that E3N88_34143, E3N88_11787 and E3N88_01311 were only highly expressed in flowers, E3N88_36012 was only highly expressed in roots, and E3N88_14925 and E3N88_15091 were only highly expressed in stems (Figure 8a), which showed a tissue-specific expression pattern for these PIN genes. Seven of the *M. micrantha* PIN genes (E3N88_14925, E3N88_15091, E3N88_45195, E3N88_40533, E3N88_19756, E3N88_03858 and E3N88_03888) showed relatively high expression levels in stems (Figure 8a), suggesting that there was functional redundancy, which may be an important factor in the rapid growth of *M. micrantha* stems. To further verify the tissue expression results, all PIN genes were selected for qRT-PCR assay. The results showed that except for E3N88_01311, which also showed a high expression level in the root, the expression of other PIN genes was almost consistent with the transcriptome results (Figure 8b).

### 2.8. Expression Patterns Analysis of M. micrantha PINs under Abiotic Stress and Hormone Treatment

**Figure 9:**
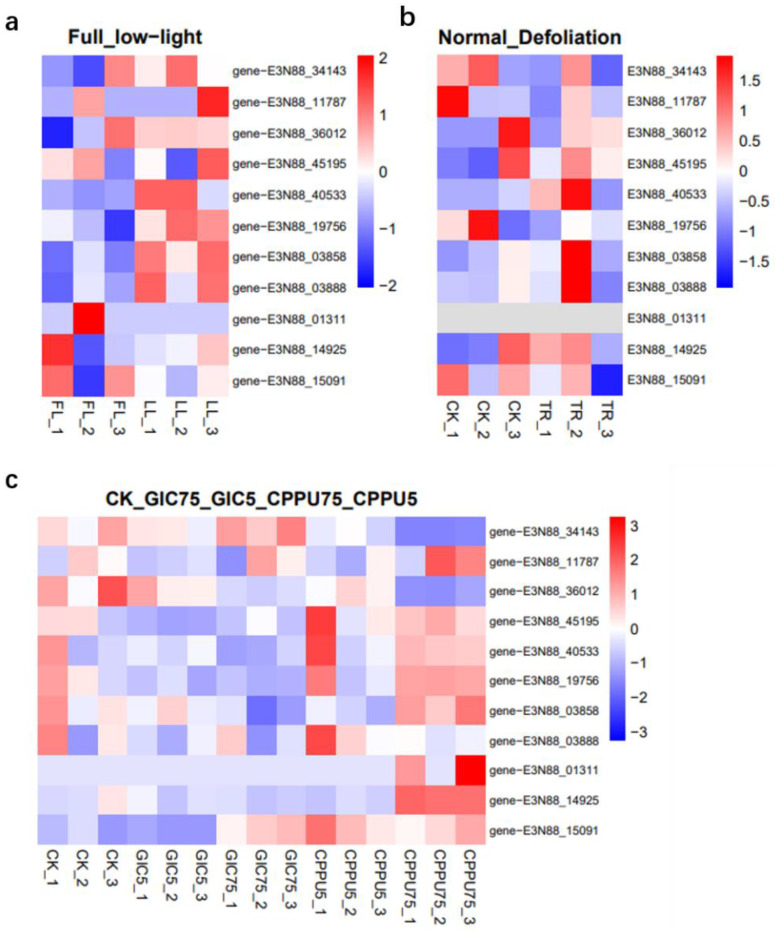
Expression heatmaps of PIN family genes under different treatments. (**a**) The expression of PIN family genes under low-light treatment. (FL = full light (100%), LL = low light (30%)). (**b**) The expression of PIN family genes of *M. micrantha* in defoliation treatment (CK = undefoliated samples, TR = defoliated samples). (**c**) The expression of PIN family genes of *M. micrantha* after CPPU or GIC treatments (CK = water application, CPPU5 and CPPU 75 = 5 ppm and 75 ppm of N-(2-Chloro-4-pyridyl)-N′-phenylurea, and GIC5 and GIC75 = 5 ppm and 75 ppm mixture of gibberellic acid, indole-3-acetic acid, and CPPU). The FPKM values were transformed to log2 (value + 1). The color scale is shown at the right, and higher expression levels are shown in red.

To understand whether the *M. micrantha* PIN genes were involved in responses under different stresses and treatments at the transcriptional level, transcriptome data were used to analyze their expression under abiotic stress (low-light, defoliation) and exogenous hormone (CPPU or GIC) treatment [35,36,40]. The results showed that under different hormone combinations, compared with the control group CK, the expression of PINs (E3N88_14925, E3N88_15091, E3N88_45195, E3N88_40533, E3N88_19756, E3N88_03858), which was high in the stem, was significantly up-regulated in the axillary buds treated with CPPU75 (Figure 9c). The expression of E3N88_40533, E3N88_19756, E3N88_03858 and E3N88_03888 in the stem was up-regulated under low-light compared with full-light conditions (Figure 9a). Under the defoliation treatment, E3N88_45195, E3N88_40533, E3N88_03858 and E3N88_03888 were up-regulated, while E3N88_19756 was down-regulated in the stems (Figure 9b). Overall, the highly expressed PIN genes in the stem showed different levels of up- or down-regulation patterns under the three stresses or treatments, suggesting that these PIN genes had an adaptive response to abiotic stress or stimuli. Among them, the expressions of E3N88_40533, E3N88_03858 and E3N88_03888 were significantly up-regulated under low light and defoliation, which was consistent with a significant increase in auxin content after treatment, indicating that these three PIN genes had an important function in the growth and stress response of *M. micrantha* stems.

## 3. Discussion

As a key regulator of plant growth and development, auxin participates in plant growth and development and in response to various environmental stresses or stimuli through polar auxin transport. Regulation of PINs at the transcriptional or post-transcriptional levels consists of spatiotemporal expression patterns, subcellular polar localization, intracellular trafficking and recycling, and degradation [8,15,18,41,42], which are used by plants to control many growth and developmental processes. Because of the publication of the whole *M. micrantha* genome, this is the first time the PIN gene family of this invasive plant has been identified and its potential roles in growth and development analyzed in response to abiotic stresses and stimuli. The results indicated that the PINs may be involved in differentiating axillary buds and adapting stems under adversity, which may be closely related to the development of axillary buds and the rapid growth of stems. At the same time, it was predicted that E3N88_40533, E3N88_03858 and E3N88_03888 may be key PIN genes involved in the rapid growth of *M. micrantha* stems and the response to environmental stresses and stimuli.

### 3.1. Identification and Evolution of the PIN Gene Family of M. mikrantha

A total of 11 PIN genes were identified in the *M. micrantha* genome, which were located on nine chromosomes (Figure 2). According to the phylogenetic analysis, the PINs of four species (*M. micrantha*, *A. thaliana*, *B. oleracea* and *V. vinifera*) were divided into six subgroups based on sequence similarity, among which canonical PINs were clustered into group I (PIN2), group V (PIN1), group VI (PIN3/4/7); noncanonical PINs were grouped into group II (PIN8) and group III (PIN5); and semi-canonical PINs were grouped into group IV (PIN6) (Figure 1). The PINs were well classified into five of these subgroups (I, III, IV, V, VI), suggesting that they may have originated from a common ancestor. The close evolutionary relationship with *A. thaliana* PINs suggested that *M. micrantha* PINs may have similar functions to the AtPINs in each subgroup. Group IV (PIN6) had a pair of paralogue genes (E3N88_14925, E3N88_15091), and group VI (PIN3/4/7) had two pairs of paralogue genes (E3N88_45195, E3N88_40533 and E3N88_03858, E3N88_03888) (Figure 1), in which their amino acid sequence homology was greater than 85%. According to the sequence similarity and the distribution on the chromosome, it was judged that the three pairs of paralogue genes were all segmental duplications (Table S2 and Table 1, Figure 2). Whole-genome data showed that the *M. micrantha* genome experienced a substantial increase in lineage-specific segmental duplications [35], and the existence of these paralogue genes may be the result of this massive duplication. Segmental, tandem, and whole-genome duplication are the main drivers for the expansion of gene families in different plant species [43]; thus, segmental duplication may be important for the amplification and evolution of *M. micrantha* PINs.

Gene amplification indicated that plant species may suffer stresses that promote the richness of this gene family or generate new functions to adapt to environmental changes [44,45]. The reason that invasive plants can successfully invade habitats is closely related to their extensive ecological adaptation. Studies have shown that *M. micrantha* tolerates low temperature, flooding and salt stress [46,47,48], has a strong photosynthetic capacity, and tolerates a wide range of light environments [49]. Further studies have found that it not only increases the photosynthetic efficiency of stems to ensure a material basis under low light and defoliation stress, but also promoted the rapid elongation of stems by synthesizing more auxin to regulate the cell length and internode length in *M. micrantha* stem [35,36,50]. The results indicated that auxin regulation plays an important role in adaptation to adversity. PINs, as key auxin transporters, were replicated and amplified to five (E3N88_45195, E3N88_40533, E3N88_19756, E3N88_03858 and E3N88_03888) in group VI (PIN3/4/7) and the number of *M. micrantha* PINs was higher than that of vines such as *V. vinifera* (0) [51], *C. lanatus* (2) [52] and *Phaseolus vulgaris* (2) [53].

At the same time, a selection pressure analysis of these duplicated genes showed that the Ka/Ks values of the two pairs of paralogue genes (E3N88_45195, E3N88_40533 and E3N88_03858, E3N88_03888) were all less than 1 (Table S3), indicating that they underwent a strong purifying or negative selection pressure with slight changes after duplication; that is, there may be functional redundancy. Therefore, the increase in the abundance of PINs in this subgroup may be the evolutionary result of *M. micrantha* in response to various environmental stresses and may help it to adapt and successfully invade through PIN-dependent auxin regulation. In the prediction of cis-acting regulatory elements, *M. micrantha* PIN genes had the highest number of light-responsive elements and were significantly higher than those of the two vines (*C. lanatus* and *V. vinifera*) (Figure 7b), which may also be compatible with high photosynthetic capacity and tolerance for a wide range of light environments.

Regarding physicochemical properties, the size and molecular weight of *M. micrantha* PIN proteins in the same subgroup were similar (Table 1) and comparable to those of *A. thaliana*, *V. vinifera* and *B. oleracea*, meaning that the PIN member structures of the same subgroup were conserved across species. Secondly, an analysis of the PIN gene intron–exon structure (Figure 3) showed that the number of exons of the *M. micrantha* PIN genes varied from one to seven, and the gene lengths were quite different, mainly due to the differences in intron lengths. Except for the PIN structure of E3N88_36012 in group I (PIN2), which was different from that of the other species, the PIN gene structure of the different species in the other subgroups tended to be conserved, which was similar to the results of the gene structure in the vine *C. lanatus* [52]. The prediction results of the transmembrane structure showed that, except for E3N88_36012, which only had a N-terminal transmembrane region, the other *M. micrantha* PINs all had transmembrane regions at both terminals and a central hydrophilic loop (Figure 4). Furthermore, a multiple sequence alignment analysis showed that the transmembrane regions at both terminals of *M. micrantha* PINs were highly conserved, and the sequence of the central hydrophilic loop was quite different (Figure 6), which was consistent with the results reported for *A. thaliana* [54]. The conserved motifs and amino acid sequence alignment of the *M. micrantha* PINs were further analyzed according to whether the central hydrophilic region contained the consensus conserved motifs 9 and 10 and the HC1–HC4 conserved region. The *M. micrantha* PINs were divided into canonical, noncanonical and semi-canonical (Figure 5 and Figure 6), and the prediction of their subcellular localization was also consistent with their taxonomic characteristics (Table 1). A further observation of the semi-canonical PINs group IV (PIN6) showed that it clustered into two clades. Unlike the clade represented by AtPIN6, *M. micrantha* E3N88_14925 and E3N88_15091 and *V. vinifera* GSVIVT01031663001 (VvPIN6b) lacked the conserved motifs 9 and 10 in the hydrophilic domain (Figure 5). This feature was close to a noncanonical PIN, which was found in other studies [51]. Moreover, the Ka/Ks ratio of this pair of paralogue genes was greater than 1 (Table S3), indicating that it underwent positive selection after replication and that functional differentiation may occur. Therefore, it was speculated that *M. micrantha* E3N88_14925 and E3N88_15091 had functional differences from *A. thaliana* AtPIN6 through the selective loss of conserved motifs in the hydrophilic region, but this needs to be confirmed by experimental studies.

### 3.2. The Expression Profile of PIN Genes in Different Tissues Predicts Its Role in the Growth and Development of M. micrantha

Some studies have demonstrated the regulatory role of PIN proteins in plant growth and development. In this regard, the expression patterns of PINs in different tissues of *M. micrantha* were analyzed (Figure 8a,b). Studies showed that *A. thaliana AtPIN1* was expressed in both vascular tissues and developmental organs and was mainly involved in shoot apical meristem circulation and flower bud formation [55]. Rice *OsPIN1c* and *OsPIN1d* were involved in panicle formation [56], and *A. thaliana AtPIN5* was involved in developmental processes such as lateral root formation and cotyledon expansion [57]. The PINs belonging to group V (PIN1) E3N88_34143, E3N88_11787 and group III (PIN5) E3N88_01311 were highly expressed in flowers, indicating that their regulatory functions in the formation and development of *M. micrantha* flower buds are conserved. Group I (PIN2) E3N88_36012 was only highly expressed in the roots and may have a similar function to that of *A. thaliana AtPIN2*, which was specifically expressed in the root apical meristem and elongation zone and participated in root gravitational growth [58].

Whether climbing and winding around other plants or covering the ground [59], the rapid growth characteristics of *M. micrantha* are inseparable from its strong branching ability. *A. thaliana* AtPIN3, -4 and -7 were usually divided into the same subgroup due to their high sequence similarity and close evolutionary relationship, and as such there was functional overlap; that is, all three were involved in negative gravitropic stem growth, phototropic plant growth and early lateral root development [13,60,61]. In addition, other functions of PIN4 in different plants were identified. *SlPIN4*-silenced plant lines in tomatoes exhibited phenotypes such as increased apical dominance, decreased axillary buds and slender stems, indicating that *SlPIN4* was involved in the establishment of plant architecture by altering auxin transport in the main stem [62]. Tobacco *NtPIN4*, highly expressed in stems and branches, regulated axillary bud germination, and mutations on the gene led to a stable and heritable abundant branching phenotype [63]. Likewise, *A. thaliana AtPIN6*-overexpressing lines increased stem branching and decreased apical dominance at the shoot tips [19]. A total of seven PIN genes—Group IV (PIN6) E3N88_14925 and E3N88_15091 and group VI (PIN3/4/7) E3N88_45195, E3N88_40533, E3N88_19756, E3N88_03858 and E3N88_03888—were highly expressed in the stem of *M. micrantha*, indicating that they may be important for stem growth and branching architecture and may be closely related to rapid growth.

### 3.3. Members of the PIN Gene Family of M. micrantha Have Different Response Modes to Abiotic Stress and Hormone Treatment

The analysis of cis-acting regulatory elements of *M. micrantha* PIN genes predicted that light, MeJA, abscisic acid and low temperature response elements were widely present in the promoter region (Figure 7b), suggesting that PINs were important for responses to abiotic stresses and environmental stimuli. Expression responses under low light, defoliation and hormone treatment showed that *M. micrantha* PINs had different response patterns under different stresses and treatments (Figure 9).

Different hormone combination treatments showed that high concentrations of CPPU (CPPU75) significantly reduced the level of auxin in the axillary buds, and affected auxin signal transduction pathways, thereby inhibiting the axillary bud differentiation [40]. However, most PIN genes were significantly up-regulated except for E3N88_34143, E3N88_36012 and E3N88_03888 (Figure 9c). It was speculated that, under CPPU75 treatment, cytokinin in the axillary buds significantly increased, and studies have shown that cytokinin promoted the accumulation of *A. thaliana* PINs on the plasma membrane [64]; therefore, the expression of PINs exhibited an opposite trend with the level of auxin. The response pattern of *M. micrantha* PINs in axillary buds under hormonal stimulation indicated that they might be involved in regulating axillary bud differentiation. At the same time, the initial development of axillary buds was a premise for determining the number of plant branches [65]. The high-level expression of most PINs in the axillary buds also indicated that the regulation of PINs may be related to the branching architecture.

*M. micrantha* showed a rapid stem elongation plasticity response to shading by significantly increasing auxin content and transcriptome level regulation under low-light stress [35]. The expression of most PINs was up-regulated compared with full light conditions, and those in group VI (PIN3/4/7) showed the same response trend (Figure 9a). PIN3/4/7 subgroups such as *A. thaliana* AtPIN3 and *Medicago truncatula* MtPIN3 were also found to be involved in plant shading responses [66,67], so it was further speculated that the *M. micrantha* PINs may promote the rapid elongation of the stem under low light by regulating the auxin level of the stem. Similarly, under defoliation stress, *M. micrantha* maintained stem growth by improving the photosynthesis rate and increasing the amount of auxin and other phytohormones [35]. Under this stress treatment, *M. micrantha* PINs also showed varying degrees of expression compared to the control group, and most of the expression in group VI (PIN3/4/7) was up-regulated (Figure 9b), which also indicated that these PINs played an important role in the response and adaptation of the stem to defoliation stress.

In conclusion, E3N88_40533, E3N88_03858 and E3N88_03888 in group VI (PIN3/4/7) were up-regulated in response to both abiotic stresses. It was speculated that *M. micrantha* may regulate its growth and stress response by regulating the expression of these PINs in the stem, which may be closely related to its strong environmental adaptability and can be used as key verification PIN genes.

## 4. Materials and Methods

### 4.1. Screening and Identification of Gene Family

To identify the possible PIN genes in *M. micrantha*, all of its protein sequence data were downloaded from the National Center for Biotechnology Information (NCBI) website. The gene identifier of *Arabidopsis AtPINs* was obtained from Yang et al. (2019), and the corresponding protein sequences were obtained as query sequences to perform a BLAST search [44]. The E value was ≤10^−8^, the identity was >60%, and the candidate genes were screened. The CD-search (https://www.ncbi.nlm.nih.gov/Structure/bwrpsb/bwrpsb.cgi, accessed on 16 August 2021) and SMART (http://smart.embl-heidelberg.de/, accessed on 16 August 2021) databases were searched to confirm the existence of the PIN-conserved domain (Pfam 03547: Mem_trans) to verify the putative PIN gene core sequence and to screen out all PIN gene sequences of *M. micrantha*. Gene ID information, chromosomal location and exon number were all obtained from *M. micrantha* gene annotation files. PASy (https://web.expasy.org/protparam/, accessed on 23 August 2021) was used to calculate the length, molecular weight (*M_W_*), isoelectric point (pI), and hydropathicity (GRAVY) analysis of the PIN proteins. The transmembrane helices of *M. micrantha* PIN proteins were predicted using TMHHMv.2.0 (https://services.healthtech.dtu.dk/service.php?TMHMM-2.0, accessed on 6 September 2021), and protein subcellular localization was predicted using WoLF PSORT (https://wolfpsort.hgc.jp/, accessed on 23 August 2021).

### 4.2. Chromosome Localization, Phylogenetic Analysis and Gene Duplication

The genome visualization tool MapChart (version 1.0.0.0) [68] was used to map the location of the PIN genes on the chromosomes. Based on their amino acid sequences, ClutalW in MEGA (version 11) software [69] was used to perform multiple sequence alignments, after which the neighbor-joining method (NJ), Poisson distances and pairwise deletion were used to construct a phylogenetic tree based on full-length sequences of *M. micrantha*, *A. thaliana*, *B. oleracea* and *V. vinifera* PIN proteins, and 1000 bootstrap replications were performed based on multiple alignments of protein sequences encoded by the PIN genes. Accession numbers of the PIN proteins are listed in Table S1. The screening criteria for duplicated gene pairs were that the length of alignable sequence covers had to be >75% of longer gene, and the similarity of aligned regions also had to be >75% [70,71]. The Ka (nonsynonymous substitution rate) and Ks (synonymous substitution rate) were investigated by using the KAKS_Calculator software (version 2.0) [72], and the selection pressure was calculated by the Ka/Ks ratio.

### 4.3. Analysis of Gene Structure, Conserved Motif and Multiple Sequence Alignment

The online program Multiple Em for Motif Elicitation (MEME) (https://meme-suite.org/meme/tools/meme, accessed on 6 September 2021) was used to analyze the conserved motifs in PIN proteins with the following settings: the maximum number of motifs was 10; minimum motif width was 6; maximum motif width was 50, and number of repetitions was arbitrary. Intron–exon structure information was included in the *M. micrantha* gif file downloaded from the NCBI. Both conserved motifs and gene structures were visualized using TBtools software (version 1.098696) [73]. The amino acid multiple sequence alignment analysis of *M. micrantha* PINs was performed using DNAMAN software (version 6.0.3.99) [74] with default parameter settings.

### 4.4. Analysis of Cis-Acting Regulatory Elements (CAREs)

The upstream sequence (2 kb) of the PIN gene was retrieved from the *M. micrantha*, *C. lanatus* and *V. vinifera* genomes by TBtools software (version 1.098696) [73] according to the gene ID, and then submitted to the PlantCare online server (http://bioinformatics.psb.ugent.be/webtools/plantcare.html, accessed on 6 September 2021) for analysis. The PIN gene ID numbers of *C. lanatus* and *V. vinifera* came from Shang et al. (2021) and Hu et al. (2021), respectively [51,52]. The number of occurrences of each CARE motif was counted in the *M. micrantha* PIN genes, and the most common CAREs were selected in TBtools, and the numbers of various response-related elements in the *M. micrantha*, *C. lanatus* and *V. vinifera* PIN genes were counted.

### 4.5. Analysis of Gene Expression Pattern

Expression profiles of PIN gene family members in different tissues/organs of *M. micrantha* were identified from the NCBI (http://www.ncbi.nlm.nih.gov/, accessed on 5 November 2021) sequence read archive (SRA) database (https://www.ncbi.nlm.nih.gov/sra, accessed on 5 November 2021) to obtain the RNA-seq dataset (accession number: no. SRR8857621-RR8857640). Similarly, the response expression profiles of PIN gene family members under different stress treatments (low light, defoliation, different phytohormone combinations) and their respective RNA-seq datasets were obtained for further analysis (low light treatment accession number: no. SRR10810948-SRR10810953; defoliation treatment accession number: no. SRR8846782-SRR8846787; different phytohormone combination treatments accession number: no. SRR12975376-SRR12975390). The downloaded raw data were subjected to quality control such as de-linking and redundancy removal to obtain clean data for subsequent comparison and quantitative analysis. The Fragments Per Kilobase of exon model per Million (FPKM) mapped fragments were used to estimate the gene expression level. Heatmaps of PIN gene family members in different tissues or under various stress/treatments were drawn using the pheatmap package in the R program (version 4.0.5) [75].

## Figures and Tables

**Table 1:** Information on PIN genes and properties of the deduced proteins in *M. micrantha*.

Gene ID	Chromosome Location (bp)	No.of Extrons	Deducted Polypeptid			GRAVY	No. of Transmembrane	Subcellular Localization
			Length (aa)	MI wt (Da)	pI			
E3N88_34143	CM018695.1: 91859336-91862569 (+)	6	572	61,745.42	8.71	0.255	8	Plasma membrane
E3N88_11787	CM018685.1: 3667162-3677387 (+)	5	573	62,411.30	9.34	0.154	8	Plasma membrane
E3N88_36012	CM018696.1: 73212228-73213496 (+)	1	422	45,883.24	9.12	−0.093	5	Plasma membrane
E3N88_45195	SZYD01002185.1: 15848-18924 (−)	6	636	69,032.71	6.43	0.164	9	Plasma membrane
E3N88_40533	CM018698.1: 55168829-55172292 (−)	6	633	69,241.85	6.07	0.111	7	Plasma membrane
E3N88_19756	CM018689.1: 58075607-58078526 (+)	4	587	63,755.81	7.75	0.164	7	Plasma membrane
E3N88_03858	CM018681.1: 5291458-5301981 (−)	7	715	77,840.88	8.01	0.040	7	Plasma membrane
E3N88_03888	CM018681.1: 6507146-6517256 (+)	7	715	77,912.95	7.64	0.049	7	Plasma membrane
E3N88_01311	CM018680.1: 78226254-78230774 (+)	5	352	38,726.75	6.51	0.725	8	Vacular membrane
E3N88_14925	CM018686.1: 59025212-59028402 (−)	4	414	45,963.12	7.89	0.353	7	Plasma membrane
E3N88_15091	CM018686.1: 63654766-63660902 (+)	6	503	56,134.80	9.10	0.122	7	Plasma membrane

## Data Availability

All datasets for this study are included in the manuscript and/or the Supplementary Materials.

## Supplementary Materials

The following supporting information can be downloaded at: https://www.mdpi.com/article/10.3390/ijms231710183/s1.
