# Supplementary material for: Genome-Wide Characterization of PIN Auxin Efflux Carrier Gene Family in Mikania micrantha
Source: Int J Mol Sci. 2022 Sep 5;23(17):10183. doi: 10.3390/ijms231710183 (PMC9456128; doi:10.3390/ijms231710183)
Supplement: Supplementary file 1 [file ijms-23-10183-s001.zip › ijms-1852165-supplementary.pdf]

Table S1. Accession numbers of the PIN genes used in phylogenetic analysis.

Organism	Assigned name	Locus ID
Mikania mikrantha		E3N88_34143
		E3N88_11787
		E3N88_36012
		E3N88_45195
		E3N88_40533
		E3N88_19756
		E3N88_03858
		E3N88_03888
		E3N88_01311
		E3N88_14925
		E3N88_15091
Arabidopsis thaliana	AtPIN1	AT1G73590.1
	AtPIN2	AT5G57090.1
	AtPIN3	AT1G70940.1
	AtPIN4	AT2G01420.2
	AtPIN5	AT5G16530.1
	AtPIN6	AT1G77110.1
	AtPIN7	AT1G23080.1
	AtPIN8	AT5G15100.1
Brassica oleracea		Bo2g012370.1
		Bo2g032760.1
		Bo2g070690.1
		Bo2g080660.1
		Bo2g134160.1
		Bo3g012090.1
		Bo6g087700.1
		Bo6g094450.1
		Bo6g112590.1
		Bo6g116850.1
		Bo6g121080.1
		Bo7g056420.1
		Bo8g071640.1
		Bo9g130710.1
		Bo9g163100.1
		Bo9g165420.1
Vitis vinifera	VvPIN1b	GSVIVT01025749001
	VvPIN1c	GSVIVT01025748001
	VvPIN2	GSVIVT01029266001
	VvPIN5a	GSVIVT01019110001
	VvPIN5b	GSVIVT01019126001
	VvPIN6a	GSVIVT01010025001
	VvPIN6b	GSVIVT01031663001
	VvPIN8	GSVIVT01011347001

Table S2. Percent amino acid sequence identity of *Mikania mikrantha* PINs.

	E3N88_34143	E3N88_11787	E3N88_36012	E3N88_45195	E3N88_40533	E3N88_19756	E3N88_03858	E3N88_03888	E3N88_01311	E3N88_14925	E3N88_15091
E3N88_34143	100	61.73	61.58	69.30	60.21	64.10	59.17	58.63	48.24	52.43	54.41
E3N88_11787	61.73	100	51.28	56.87	57.45	58.18	56.69	56.51	53.75	44.44	43.19
E3N88_36012	61.58	51.28	100	53.92	54.71	59.95	51.79	51.79	46.47	53.27	52.80
E3N88_45195	69.30	56.87	53.92	100	85.14	79.41	73.68	73.34	50.00	53.16	51.27
E3N88_40533	60.21	57.45	54.71	85.14	100	77.19	67.38	65.79	49.41	53.16	50.11
E3N88_19756	64.10	58.18	59.95	79.41	77.19	100	80.38	80.56	49.41	51.97	51.72
E3N88_03858	59.17	56.69	51.79	73.68	67.38	80.38	100	98.46	53.85	51.85	48.02
E3N88_03888	58.63	56.51	51.79	73.34	65.79	80.56	98.46	100	53.85	52.35	48.12
E3N88_01311	48.24	53.75	46.47	50.00	49.41	49.41	53.85	53.85	100	47.38	44.54
E3N88_14925	52.43	44.44	53.27	53.16	53.16	51.97	51.85	52.35	47.38	100	97.83
E3N88_15091	54.41	43.19	52.80	51.27	50.11	51.72	48.02	48.12	44.54	97.83	100

Table S3. Analysis of selection pressure of three pairs of paralogue PIN genes in *Mikania mikrantha*.

Sequence	Method	Ka	Ks	Ka/Ks	P-Value(Fisher)	Length	S-Sites	N-Sites	Fold-Sites(0:2:4)	Substitutions	S-Substitutions	N-Substitutions	Fold-S-Substitutions(0:2:4)	Fold-N-Substitutions(0:2:4)	Divergence-Time	Substitution-Rate-Ratio(rTC:rAG:rTA:rCG:rTG:rCA/rCA)	GC(1:2:3)	ML-Score	AICc	Akaike-Weight	Model
E3N88_40533&E3N88_45195	MA	0.0869297	1.01257	0.0858507	1.05E-142	1860	445.667	1414.33	NA	361	283.705	77.2951	NA	NA	0.308718	1.627:1.69454:1.27491:1.28432:0.60994:1	0.479296(0.493012:0.42236:0.522516)	-3555.44	NA	NA	NA
E3N88_03858&E3N88_03888	MA	0.0075336	0.0525347	0.143402	5.12E-09	2145	485.781	1659.22	NA	37	24.8355	12.1645	NA	NA	0.0177251	3.35566:3.80592:3.73909:3.68658:3.83479:1	0.507216(0.509078:0.440642:0.571927)	-3047.49	NA	NA	NA
E3N88_14925&E3N88_15091	MA	1.01735	0.941911	1.08009	0.0735547	1242	285.627	956.373	NA	886	191.921	694.079	NA	NA	1	0.863556:0.857664:0.931516:0.924674:1.17899:1	0.364811(0.402584:0.361829:0.33002)	-3405.72	NA	NA	NA
